# Von Hippel-Lindau (*VHL*) Inactivation in Sporadic Clear Cell Renal Cancer: Associations with Germline *VHL* Polymorphisms and Etiologic Risk Factors

**DOI:** 10.1371/journal.pgen.1002312

**Published:** 2011-10-13

**Authors:** Lee E. Moore, Michael L. Nickerson, Paul Brennan, Jorge R. Toro, Erich Jaeger, Jessica Rinsky, Summer S. Han, David Zaridze, Vsevolod Matveev, Vladimir Janout, Hellena Kollarova, Vladimir Bencko, Marie Navratilova, Neonilia Szeszenia-Dabrowska, Dana Mates, Laura S. Schmidt, Petra Lenz, Sara Karami, W. Marston Linehan, Maria Merino, Stephen Chanock, Paolo Boffetta, Wong-Ho Chow, Frederic M. Waldman, Nathaniel Rothman

**Affiliations:** 1 Division of Cancer Epidemiology and Genetics, National Cancer Institute, National Institutes of Health, Bethesda, Maryland, United States of America; 2 Cancer and Inflammation Program, National Cancer Institute, Frederick, Maryland, United States of America; 3 International Agency for Research on Cancer, Lyon, France; 4 University of California San Francisco, San Francisco, California, United States of America; 5 Institute of Carcinogenesis, Cancer Research Centre, Moscow, Russia; 6 Department of Preventive Medicine, Faculty of Medicine, Palacky University, Olomouc, Czech Republic; 7 Institute of Hygiene and Epidemiology, Charles University, Prague, Czech Republic; 8 Department of Cancer Epidemiology and Genetics, Masaryk Memorial Cancer Institute, Brno, Czech Republic; 9 Department of Epidemiology, Institute of Occupational Medicine, Lodz, Poland; 10 Institute of Public Health, Bucharest, Romania; 11 Basic Science Program, SAIC–Frederick, NCI–Frederick, Frederick, Maryland, United States of America; 12 Urologic Oncology Branch, National Cancer Institute, National Institutes of Health, Bethesda, Maryland, United States of America; 13 Support to the Division of Cancer Epidemiology and Genetics, National Cancer Institute, SAIC-Frederick, NCI- Frederick, Frederick, Maryland, United States of America; 14 Core Genotyping Facility at the Advanced Technology Center of the National Cancer Institute, National Institutes of Health, Department of Health and Human Services, Bethesda, Maryland, United States of America; 15 The Tisch Cancer Institute, Mount Sinai School of Medicine, New York, New York, United States of America; 16 International Cancer Prevention Research Institute, Lyon, France; University of Birmingham, United Kingdom

## Abstract

Renal tumor heterogeneity studies have utilized the von Hippel-Lindau *VHL* gene to classify disease into molecularly defined subtypes to examine associations with etiologic risk factors and prognosis. The aim of this study was to provide a comprehensive analysis of *VHL* inactivation in clear cell renal tumors (ccRCC) and to evaluate relationships between *VHL* inactivation subgroups with renal cancer risk factors and *VHL* germline single nucleotide polymorphisms (SNPs). *VHL* genetic and epigenetic inactivation was examined among 507 sporadic RCC/470 ccRCC cases using endonuclease scanning and using bisulfite treatment and Sanger sequencing across 11 CpG sites within the *VHL* promoter. Case-only multivariate analyses were conducted to identify associations between alteration subtypes and risk factors. *VHL* inactivation, either through sequence alterations or promoter methylation in tumor DNA, was observed among 86.6% of ccRCC cases. Germline *VHL* SNPs and a haplotype were associated with promoter hypermethylation in tumor tissue (OR = 6.10; 95% CI: 2.28–16.35, p = 3.76E-4, p-global = 8E-5). Risk of having genetic *VHL* inactivation was inversely associated with smoking due to a higher proportion of wild-type ccRCC tumors [former: OR = 0.70 (0.20–1.31) and current: OR = 0.56 (0.32–0.99); P-trend = 0.04]. Alteration prevalence did not differ by histopathologic characteristics or occupational exposure to trichloroethylene. ccRCC cases with particular *VHL* germline polymorphisms were more likely to have *VHL* inactivation through promoter hypermethylation than through sequence alterations in tumor DNA, suggesting that the presence of these SNPs may represent an example of facilitated epigenetic variation (an inherited propensity towards epigenetic variation) in renal tissue. A proportion of tumors from current smokers lacked *VHL* alterations and may represent a biologically distinct clinical entity from inactivated cases.

## Author Summary

In a large case-series of 470 sporadic clear cell renal cancer (ccRCC) cases, we examined von Hippel-Lindau (*VHL*) inactivation as a biomarker of tumor heterogeneity. Germline alterations of the *VHL* gene were identified and have been found in most families with VHL disease, a hereditary syndrome associated with ccRCC. In sporadic disease, *VHL* alterations have been reported in up to 91% of cases. Here, we observed a high prevalence of *VHL* inactivation through both genetic and epigenetic mechanisms that were highly associated with ccRCC. *VHL* inactivation through promoter hypermethylation in tumors was associated with inherited polymorphisms selected to capture common variation across the *VHL* locus. A high-risk haplotype associated with promoter hypermethylation in tumor DNA was identified. These findings suggest that the presence of these polymorphisms and *VHL* promoter hypermethylation may represent an example of an inherited propensity toward epigenetic variation and potential silencing of the *VHL* gene in tumor tissue. This result could have translational implications, as individuals with the high-risk haplotype could be targeted for increased surveillance. Smokers had a higher prevalence of tumors without detectable *VHL* sequence alteration or epigenetic inactivation. Such tumors may be biologically distinct and have demonstrated a poorer prognosis compared to *VHL* inactivated cases.

## Introduction

Von Hippel-Lindau *(VHL)* alteration leading to protein inactivation is considered a frequent, early event in renal carcinogenesis that can be used as a biomarker of tumor heterogeneity, to strengthen etiologic relationships with risk factors, and study mechanistic pathways of disease [1]–[4]. The most common established risk factors that are associated with approximately 50% of renal cell cancer (RCC) cases include obesity, hypertension, and tobacco smoking. Less-established risk factors include occupational exposure to pesticides and the organic solvent trichloroethylene (TCE). Dietary intake of vegetables and fruits has been inversely associated with renal cancer, whereas intake of red meat and milk products have been associated with increased RCC risk, although not consistently [5]. Common genetic variation has also been shown to modify RCC risk [5].

Germline sequence alterations of the *VHL* gene were first identified and have been observed in almost all families with VHL disease, a hereditary cancer syndrome in which affected individuals are at risk for renal cysts and clear cell RCC (ccRCC) [1]. In sporadic ccRCC, alterations in the *VHL* gene have been reported in up to 91% of case tumors [6]. The *VHL* gene plays a role in tissue-specific responses to oxygen concentration and delivery. Under normal oxygen conditions, the VHL protein forms a complex with elongin B, elongin C, and cullin 2 which targets hydroxylated hypoxia inducible factor-alpha (HIFα) for ubiquitin-mediated degradation [7], [8]. Under hypoxic conditions, the VHL complex cannot bind HIFα for degradation because it is in the non-hydroxylated form. Therefore, HIFα accumulates, resulting in transcription of additional genes that facilitate oxygen delivery, cellular adaptation to oxygen deprivation, and angiogenesis. Similarly, alteration of the *VHL* gene prevents formation of the protein complex required for HIF degradation, resulting in an excess of HIF and a similar gene expression pattern as that observed under hypoxic conditions.

Recently, we applied sensitive and high-throughput mutation detection methods in a pilot study of 205 well-characterized sporadic ccRCC cases to comprehensively evaluate tumor DNA for *VHL* sequence alterations and promoter methylation, and to identify associations between the prevalence, type, and location of *VHL* alterations with etiologic and clinical factors [6]. In the current study, we expand upon our previous analysis [6], and report findings from the entire case series of 507 sporadic RCC cases, including 470 ccRCC patients. The combined dataset has increased statistical power to identify associations between risk factors and heterogeneous subgroups of cases defined by the type of *VHL* alterations. Using questionnaire data on patient characteristics, nutritional intake, and occupational exposures known or suspected to modify risk, we attempted to replicate findings from previously published studies in which evidence of tumor heterogeneity had been reported [9]–[13]. A previously reported *VHL* polymorphism, rs779805 and epigenetic inactivation of the *VHL* gene were examined among these cases using a tag SNPs method that is routinely used in association studies. The *VHL* gene locus was examined using highly correlated SNPs to comprehensively evaluate common genetic variation across this gene region and risk of developing a specific type of *VHL* alteration [14].

## Results

### Patient and Tumor Characteristics

In Table S1, sporadic RCC cases from the Central and Eastern European Case-Control study (CEERCC) that were included in this analysis are compared to cases not included in this analysis by their personal/clinical characteristics, and risk factors that have been previously associated with the prevalence of RCC or *VHL* alterations in tumor tissue. Cases not included in this study were those from whom we were unable to collect frozen tumor biopsy material for analyses. Collection of frozen tissue was most successful among cases from the Czech Republic than the other study regions. This analysis also included more cases with high body mass index (BMI), with a lower level of education, and included more M0 compared to M1 cases compared to those not included in this analysis. The distribution of other factors was similar across groups.

### *VHL* Alteration Prevalence in RCC and ccRCC Tumor DNA

The prevalence of having any *VHL* alteration (i.e. inactivating, silent, and intronic mutations, and promoter hypermethylation) was 83.0% among all RCC cases, 88.3% among the subset of ccRCC cases, and 16.2% among non-ccRCC cases (Table 1). The higher percentage observed in ccRCC compared to all RCC cases was due to a greater proportion of cases with *VHL* inactivating alterations and a lower proportion of wild type ccRCC cases [i.e. cases without inactivating *VHL* gene alterations], that were primarily observed among non-ccRCC cases. The overall prevalence of *VHL* promoter hypermethylation was similar in RCC, ccRCC, and non-ccRCC cases. Among all ccRCC cases, the highest alteration prevalence was located at codon 117 (N = 13). These included 5 deletions, 5 missense, 2 insertions, and 1 nonsense sequence alteration at Codon 65 had the second highest sequence alteration prevalence (N = 11), and included 6 missense, 4 nonsense, and one deletion (data not shown). Thirty putative splice junction sequence alterations, (intronic variants located within 3 bases of an exon) were observed. *VHL* promoter epigenetic and genetic inactivation was mutually exclusive in all tumors analyzed. Because *VHL* genetic alterations were rarely observed among non-ccRCC cases, all additional analyses were restricted to confirmed ccRCC cases.

**Table 1:** *VHL* gene alterations among histologically confirmed RCC, clear cell RCC, and non-clear cell RCC cases.

	All RCC Cases (N = 507)		Clear Cell RCC (N = 470)		Non clear cell RCC (N = 37)	
VHL Status	N	%	N	%	N	%
**Any Alteration**	421	83.0%	415	88.3%	6	16.2%
**Functional Alteration**	368	72.6%	366	77.9%	2	5.4%
**1 Alteration**	352	69.4%	350	74.5%	2	5.4%
**2 Alterations**	14	2.8%	14	3.0%	0	0.0%
**3 Alterations**	2	0.4%	2	0.4%	0	0.0%
**No Functional Genetic Alteration**	139	27.4%	104	22.1%	35	94.6%
**P25L**	13	2.6%	12	2.6%	1	2.7%
**Silent**	13	2.6%	13	2.8%	0	0.0%
**Precodon 54**	3	0.6%	3	0.6%	0	0.0%
**Methylated**	44	8.7%	41	8.7%	3	8.1%

	Total Alterations (N = 386)		Total Alterations (N = 384)		Total Alterations (N = 2)	
Type of Genetic Alteration	N	%	N	%		
**Deletion**	146	37.8%	145	37.8%	1	50.0%
**Insertion**	62	16.1%	62	16.1%	0	0.0%
**Missense**	107	27.7%	107	27.9%	0	0.0%
**Nonsense**	39	10.1%	39	10.2%	0	0.0%
**Splicing**	31	8.0%	30	7.8%	1	50.0%
**3′ UTR**	1	0.3%	1	0.3%	0	0.0%
**Total**	386	100.0%	384	100.0%	2	100.0%

Location	N	%	N	%	N	%
**Exon 1**	141	36.5%	141	36.7%	0	0.0%
**Intron 1**	8	2.1%	8	2.1%	0	0.0%
**Exon 2**	116	30.1%	115	29.9%	1	50.0%
**Intron 2**	21	5.4%	20	5.2%	1	50.0%
**Exon 3**	98	25.4%	98	25.5%	0	0.0%
**Total**	384	99.5%	382	99.5%	2	100.0%

RCC include clear cell, papillary, chromophobe cases. ccRCC includes clear cell, ccRCC with papillary features, and sarcomatoid differentiation.

Non ccRCC includes papillary and chromophobe RCC cases.

Any alteration group includes *VHL* inactivating and non-inactivating genetic alterations and promoter hypermethylation.

Non functional genetic alteration group includes subjects without *VHL* inactivating alterations.

Total number of mutation group lacks the location of two splices with unknown intron location.

### Univariate Analysis of *VHL* Alteration Subtypes by Tumor and Patient Characteristics in ccRCC

From the univariate analysis (Table S2), risk factors with a p-value<0.20 were selected as adjustment variables in multivariate analyses. In addition to sex, age, and country, variables remained in models if their inclusion changed risk estimates by at least 10%. High blood pressure (p = 0.16), family history of cancer (p = 0.12), and fruit intake frequency (p = 0.09), were inversely associated with *VHL* promoter hypermethylation and were selected for initial inclusion in multivariate models. In addition to smoking (p = 0.05), family history of cancer (p = 0.10) and fruit intake (p = 0.14) were initially included the multivariate analysis of genetic inactivation prevalence.

In M1 compared to M0 cases, a lower prevalence of genetic inactivation was observed in exon 1 [from 43.4% to 11.8%, p = 0.02] and a higher prevalence was observed in exon 3 [from 25.4% to 52.9%, p = 0.08]. Inactivating *VHL* alterations were found in 9/12 (75%) patients with a self-reported family history of kidney cancer; 8 case tumors had inactivating alterations, and 1 had a hypermethylated *VHL* promoter. Specifically, two cases had deletions located at codons 137 and 180, two had insertions located at codons 184 and 203, and four had missense mutations, two A/G transitions at codons 120 and 147, one T/A transversion at codon 158, and one C/A transversion at amino acid 65 which resulted in a stop (ATG) codon (data not shown). Of the three *VHL* wild type cases, one had an alteration within intron 2 (-11) that was not considered as an inactivating alteration in this study.

### Multivariate Analyses

In Table 2, Odds Ratios (OR) and 95% confidence intervals (CI) estimated from multivariate models of risk factors and *VHL* alteration prevalence among ccRCC case tumor DNAs are presented. Only tobacco smoking and fruit intake, were significantly associated with *VHL* genetic inactivation in tumor tissue, and/or were required for adjustment. Tobacco smoking was inversely associated with VHL genetic inactivation in ccRCC tumors. The OR associated with having a ccRCC tumor with a genetic *VHL* inactivating alteration decreased in a dose-dependent manner among former [OR = 0.70(0.20–1.31)] and current smokers [OR = 0.56(0.32–0.99)] compared to never smokers (p-trend = 0.04). The association in the adjusted analysis was very similar to that observed in the univariate analysis as was the association with fruit intake that was not statistically significant (Table S2). The association with fruit intake and genetic alterations in tumor DNA did not differ with respect to smoking status. The lower prevalence of genetically inactivated cases among current smokers was due to a higher prevalence of wild type cases (∼8%) (Table 2). Low fruit intake frequency was associated with having a *VHL* promoter hypermethylated tumor (p = 0.02). Compared to cases with high fruit intake frequency, the association with promoter hypermethylation increased in a dose-dependent manner among cases with medium [OR = 1.37(0.62–3.03)] and low fruit intake [OR = 3.03(1.16–8.33); p-trend = 0.02]. After stratification by smoking status (ever/never), the association between fruit intake and promoter hypermethylation was observed among smokers when the high intake group was used as a referent [medium: OR = 1.72(0.59–3.13), and low: OR = 4.35(1.09–16.67); p-trend = 0.03]. A significant trend was not observed among never smokers (p-trend = 0.59), however, the formal test for heterogeneity between models was not statistically significant (p-interaction = 0.28). In contrast, tumor histopathologic factors that are generally associated with disease progression (i.e. stage, nuclear grade, node positivity, metastases) were not associated with alteration prevalence. Likewise, occupational exposure to the solvent TCE was not associated with *VHL* alteration prevalence in tumor DNA, when compared to unexposed cases.

**Table 2:** Multivariate analysis of *VHL* inactivating alterations and ccRCC risk factors.

Patient Characteristics	Subgroup	Number of Cases with/without alteration	OR-adj	(95% CI)	p-value
			**Inactivating Genetic Alterations**		
**Tobacco Smoking**	Never	172/39	1.00	Ref	
	Former	82/24	0.70	(0.37–1.31)	0.26
	Current	110/41	0.56	(0.32–0.99)	0.05
***p-trend***					***0.04***
	Ever		0.60	(0.35–1.02)	0.06
**Tertile Fruit Intake**	High	121/30	1.00	Ref	
	Medium	133/34	0.96	(0.53–1.74)	0.90
	Low	96/36	0.60	(0.33–1.10)	0.10
***p-trend***			0.77	(0.57–1.05)	0.09

			Promoter Hypermethylation		
**Tertile Fruit Intake**	High	15/108	1.00	Ref	
	Medium	17/137	1.37	(0.62–3.03)	0.44
	Low	8/125	3.03	(1.16–8.33)	0.02
***p-trend***					***0.02***
**Tertile Fruit Intake**	HIgh	5/61	1.00	Ref	
**Never Smokers**	Medium	7/61	0.92	(0.27–3.13)	0.89
	Low	5/47	1.41	(0.38–5.26)	0.61
***p-trend***					***0.59***
**Tertile Fruit Intake**	High	3/64	1.00	Ref	
**Ever Smokers**	Medium	10/76	1.72	(0.60–5.00)	0.32
	Low	10/61	4.35	(1.09–16.67)	0.04
***p-trend***					***0.03***

Tobacco Smoking analyses adjusted for age, sex, country, tertile of fruit frequency intake.

Fruit intake analyses adjusted for age, sex, country and smoking status (ever, never).

Analyses of fruit intake stratified by smoking status are adjusted for age, sex, country.

Inactivating genetic alterations: cases with deletions, insertions, missense, nonsense, splice site alterations.

In Table 3, the association between chromosome 3p loss and specifically the 3p clone CTB110j24, which harbors the *VHL* gene locus, were analyzed with respect to *VHL* gene inactivation through either epigenetic or sequence alterations. The proportion of both chromosome 3p loss, and loss of clone CTB110j24 were significantly higher among cases with *VHL* gene inactivation compared to cases without *VHL* inactivation (92.4%/93.9% vs 70.2%/61.7%; p<0.00001). Visual examination of each array CGH profile among cases without and with *VHL* inactivation but for whom chromosome 3p loss was observed, did not differ with respect to loss of clone CTB110j24, and did not support the hypothesis that biallelic loss of the *VHL* locus had occurred among cases without *VHL* inactivation (i.e. wild type cases). In adjusted multivariate analysis, the only factor that was significantly associated with chromosome 3p loss was having a *VHL* gene alteration, and the association was highly significant (OR = 1250; 95% CI:476–3125, p<0.00001). Additional analysis of array CGH data did not indicate that loss of chromosome 3p or clone CTB110j24 differed significantly in tumor DNA from never (92.1%), former (91.3%), or current smoking cases (92.3%), nor did we observe a significant trend with tobacco exposure (data not shown).

**Table 3:** Cases with/without *VHL* genetic or epigenetic inactivation and chromosome 3p and *VHL* locus loss (clone CTB-110j24) using array CGH.

	Cases without *VHL* inactivation in tumor DNA	(%)	Proportion of cases	Case with *VHL* gene inactivation in tumor DNA	(%)	Proportion of cases	Total number of cases	p-value
**3p loss**								
**No**	14	3.4	29.8	28	6.7	7.6		
**Yes**	33	8.0	70.2	340	81.9	92.4	415	p<0.00001
**CTB110j24**								
**No**	18	4.4	38.3	22	5.3	6.1		
**Yes**	29	7.0	61.7	340	83.1	93.9	409	p<0.00001

Cases without VHL inactivation Include those without genetic (sequence alterations) or epigenetic (promoter hypermethylation) of *VHL* in tumor DNA.

p-values adjusted for center, age, sex.

Comparative genomic hybridization (CGH).

### Germline *VHL* Polymorphisms and *VHL* Promoter Hypermethylation in Tumor Tissue DNA

**Figure 1:**
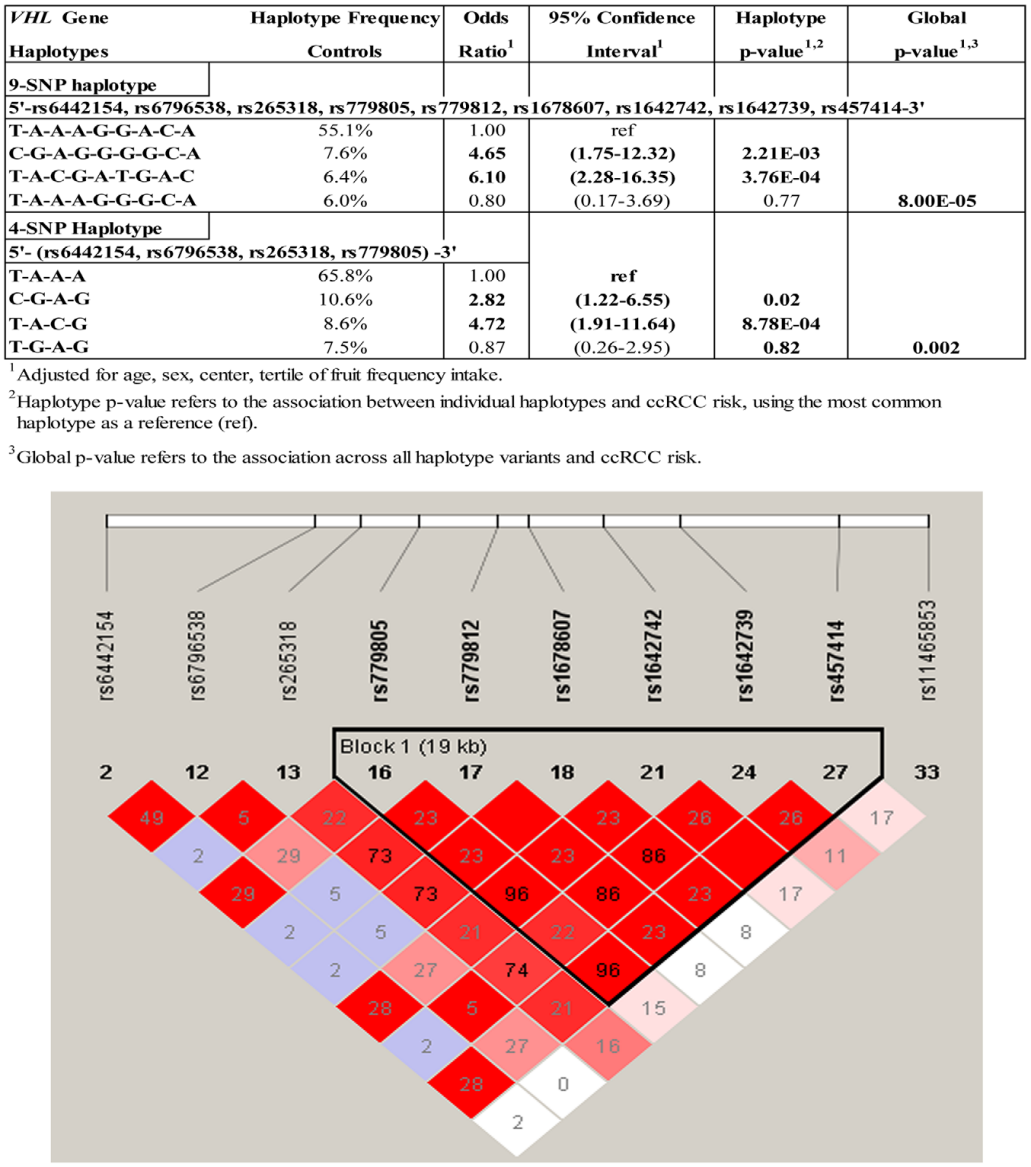
Risk of *VHL* promoter hypermethylation associated with inherited *VHL* gene polymorphisms. A haploview plot showing the correlation (r2) between the *VHL* SNPs genotyped. The number in each square represents the value of pair wise r^2^ between any two markers.

In Table 4, associations between germline *VHL* tag SNPs and *VHL* gene promoter hypermethylation in tumor tissue were evaluated in an attempt to replicate a previously reported association between the presence of *VHL* SNP rs779805 (Ex1+19G>A) in germline DNA, and the prevalence of promoter hypermethylation in tumor DNA [15]. A tag-SNP method was used that relies upon linkage disequilibrium (LD) of highly correlated SNPs to identify gene regions that could be associated with disease susceptibility, or heterogeneous subgroups of cases [14]. A significant association was observed between cases with *VHL* promoter hypermethylation with SNP rs779805, and six additional tag SNPs across the *VHL* gene region. Some associations between SNPs in germline and promoter hypermethylation in tumor DNA were stronger when analyses were restricted to ccRCC cases. Significant trends were observed in ccRCC cases that had germline minor alleles at *VHL* SNPs rs265318 (−2872 A>G), rs779805 (Ex1 +19 G>A), rs779812 (IVS1 −1184 G>A), rs1678607 (IVS2 +108 T>G), rs1642742 (Ex3 +473 G>A), rs1642739 (3835 bp 3′ST), and rs457414 (*IRAK2* −3754 A>C). Examination of this region using Haploview revealed that some tag SNPs were highly correlated (Figure 1). To identify regional associations between germline variants with promoter hypermethylation in tumor DNA, a sliding window analysis was conducted with a fixed window size ranging from 2 to 9 consecutive SNPs. The most significant global association across all inherited haplotype variants and tumor-specific *VHL* promoter hypermethylation was observed when a 9-SNP window spanning across the entire *VHL* gene region was evaluated. This window spanned from tag SNP rs6442154 (*c3orf10*, Ex3 −90, T>C) through rs457414 (*IRAK2* −3754 A>C) (p-global = 8E-5). When specific *VHL* haplotypes were evaluated individually and compared to the common 9-SNP referent haplotype T-A-A-A-G-G-A-C-A, two germline haplotypes were significantly associated with tumor-specific promoter hypermethylation, specifically T-A-C-G-A-T-G-A-C (OR = 6.10; 95% CI:2.28–16.35, p = 3.76E-4) and C-G-A-G-G-G-G-C-A (OR = 4.65; 95% CI:1.75–12.32; p = 2.21E-3, respectively). A similar association was observed with a 4-SNP haplotype, as rs779805 was able capture common variation across a highly correlated 6-SNP block, spanning from rs779805 through rs457414. Haplotype analyses spanning a 3-SNP sliding window, conducted to capture smaller regional variations, suggested that two regions may be driving the association observed, one centered within the *VHL* promoter at rs265318 (p = 0.005) and the other located 3′ of the *VHL* stop codon centered at rs457414 (p = 0.0004) (data not shown). No associations were observed between these inherited polymorphisms and ccRCC cases with genetically inactivated *VHL* or wild type tumors.

**Table 4:** Tagging SNP analysis of common *VHL* polymorphisms in genomic DNA and promoter hypermethylation in somatic tissue from ccRCC cases.

				Clear Cell RCC Cases						
*Gene*	db SNP	Location	Base Change	Unmethylated	(%)	Methylated	(%)	Odds Ratio ccRCC	(95% CI)	p-value
***c3orf10***	rs6442154	Ex3 −90	TT	236	80.3	22	68.8	REF		
			CT	56	19.1	8	25.0	1.58	(0.62–4.04)	0.34
			CC	2	0.7	2	6.3	10.10	(1.17–86.6)	0.03
			*Per allele-p-trend*					*2.08*	*(1.00–4.39)*	*0.05*
			CT+CC	58	19.8	10	31.3	1.92	(0.80–4.60)	0.14
***VHL***	rs6796538	−5011 A>G	AA	176	59.9	13	41.9	REF		
			AG	105	35.7	16	51.6	1.84	(0.82–4.15)	0.14
			GG	13	4.4	2	6.5	1.97	((0.37–10.5)	0.43
			*Per allele-p-trend*					*1.61*	*(0.86–3.01)*	*0.14*
			AG+GG	118	40.1	18	58.1	1.86	(0.85–4.07)	0.12
***VHL***	rs265318	−2872 A>C	AA	243	83.2	22	68.8	REF		
			AC	48	16.4	9	28.1	2.74	(1.12–6.74)	0.03
			CC	1	0.3	1	3.1	31.33	(1.30–756.9)	0.03
			*Per allele-p-trend*					*3.20*	*(1.43–7.15)*	*0.004*
			AC+CC	49	16.7	10	31.2	3.08	(1.29–7.37)	0.01
***VHL***	rs779805	Ex1 +19 G>A	AA	149	50.5	12	37.5	REF		
			AG	131	44.4	13	40.6	1.17	(0.48–2.81)	0.73
			GG	15	5.1	7	21.9	6.04	(1.93–18.9)	0.002
			*Per allele-p-trend*					*2.10*	*(1.16–3.79)*	*0.01*
			AG+GG	146	49.5	20	62.5	1.71	(0.77–3.79)	0.19
***VHL***	rs779812	IVS1 −1184	GG	240	81.6	22	68.8	REF		
			AG	52	17.7	9	28.1	2.58	(1.06–6.37)	0.04
			AA	2	0.7	1	3.1	6.60	(0.45–96.3)	0.17
			*Per allele-p-trend*					*2.58*	*(1.19–5.58)*	*0.01*
			AG+AA	54	18.4	10	31.2	2.76	(1.16–6.65)	0.02
***VHL***	rs1678607	IVS2 +108T>G	GG	240	81.9	22	68.8	REF		
			GT	51	17.4	9	28.1	2.58	(1.06–6.30)	0.04
			TT	2	0.7	1	3.1	6.61	(0.45–97.6)	0.17
			*Per allele-p-trend*					*2.58*	*(1.19–5.57)*	*0.02*
			GT+TT	53	18.1	10	31.2	2.76	(1.16–6.53)	0.02
***VHL***	rs1642742	Ex3 +473 G>A	AA	143	48.8	13	40.6	REF		
			AG	124	42.3	12	37.5	1.26	(0.52–3.07)	0.61
			GG	26	8.9	7	21.9	4.68	(1.52–14.4)	0.007
			*Per allele-p-trend*					*1.97*	*(1.10–3.50)*	*0.02*
			AG+GG	150	51.2	19	59.4	1.75	(0.78–3.90)	0.17
***VHL***	rs1642739	3835 bp 3′ST	CC	231	80.8	21	67.7	REF		
			AC	52	18.2	9	29.0	2.59	(1.05–6.41)	0.04
			AA	3	1.1	1	3.2	3.49	(0.30–40.3)	0.32
			*Per allele-p-trend*					*2.30*	*(1.09–4.88)*	*0.03*
			AC+AA	55	19.3	10	32.2	2.66	(1.11–6.38)	0.03
***IRAK2***	rs457414	−3754 A>C	AA	144	49.0	10	31.3	REF		
			AC	125	42.5	15	46.9	2.05	(0.83–5.08)	0.12
			CC	25	8.5	7	21.9	5.98	(1.87–19.2)	0.003
			*Per allele-p-trend*					*2.39*	*(1.33–4.30)*	*0.004*
			AC+CC	150	51.0	22	68.8	2.60	(1.11–6.09)	0.03
***IRAK2***	rs11465853	IVS1+249 G>C	GG	131	44.9	9	28.1	REF		
			GC	118	40.4	17	53.1	2.52	(1.02–6.22)	0.05
			CC	43	14.7	6	18.8	2.28	(0.69–7.60)	0.18
			*Per allele-p-trend*					*1.62*	*(0.94–2.77)*	*0.08*
			GC+CC	161	55.1	23	71.9	2.46	(1.04–5.84)	0.04

Odds ratios are adjusted for age, sex, country, and tertile of fruit frequency intake.

## Discussion

The prevalence of *VHL* inactivation in ccRCC tumor DNA from this large well-characterized case-series of sporadic RCC is one of the highest reported in the literature, and is consistent with recent publications including our previous report [6], [9], [13], [15], [16]. As in our previous study, *VHL* inactivation in ccRCC tumors occurred either through genetic or epigenetic mechanisms. Notably, *VHL* alteration prevalence was associated uniquely with etiologic risk modifiers such as tobacco use, fruit intake, and *VHL* tag SNPs that were examined to capture common germline genetic variation across this region. We observed a slightly higher prevalence of tumors without genetic or epigenetic inactivation of *VHL* gene among current and former smokers, compared to never smokers. The addition of array CGH analysis of this same case-series of chromosome 3p loss and specifically the clone that harbors the *VHL* locus, clone CTB110j24, enabled us to evaluate the *VHL* gene region for evidence of biallelic loss among cases in which we did not detect sequence alterations or evidence of epigenetic inactivation. Multivariate analysis of chromosome 3p loss revealed that the only patient/tumor characteristic or RCC risk factor associated with 3p or clone CTB110j24 loss was inactivation of the *VHL* gene, and the association was highly significant. In addition, statistically significant associations between *VHL* promoter hypermethylation and low fruit intake frequency were observed, particularly in tumor DNA from smokers. Interestingly, associations were observed with several tag SNPs spanning the *VHL* gene in germline DNA and tumor-specific *VHL* promoter hypermethylation. Of the ten tag SNPs selected to capture common genetic variation across the *VHL* gene region and evaluate associations with heterogeneous case subgroups, seven were significantly associated with *VHL* promoter hypermethylation in tumors. Analysis of haplotypes revealed the strongest association was observed using a 9-SNP sliding window that spanned across the entire *VHL* gene region. Lastly, we did not observe an increased prevalence of inactivating alterations, multiple mutations, nor a specific “hot spot” among TCE exposed cases, compared to unexposed cases.

The association between inherited *VHL* polymorphisms and promoter hypermethylation in sporadic ccRCC case tumor DNA observed in the current study was similar to a previous report of 97 sporadic RCC cases [15] in which germline SNP rs779805 (Ex1 +19 A>G) was significantly associated with tumor-specific *VHL* promoter hypermethylation. In the current study, along with rs779805, nine additional SNPs were selected to capture (tag) common variation across the *VHL* gene region to evaluate associations with heterogeneous subgroups of cases. The global p-values indicated that germline variation across the *VHL* gene region was significantly associated with risk of having a *VHL* hypermethylated tumor. When compared to the most common SNP or haplotype as a common referent, subsequent analyses revealed that several individual SNPs and two high risk haplotypes were associated with tumor-specific *VHL* promoter hypermethylation. These findings are similar to several reports of other cancer types, in which associations between constitutional (germline) mutations were associated with gene specific hypermethylation and silencing in tumors. In one report, a *MGMT* germline polymorphism (rs16906252 C>T) located within the transcriptional enhancer element of the *MGMT* promoter was strongly associated with susceptibility to CpG island methylation and gene silencing in colorectal cancer (OR = 18.0; 95% CI:6.2–52.1, p<.0001) [17], [18]. Epigenetic silencing and transcriptional suppression of the death associated protein kinase 1 gene *DAPK1*, an underlying factor in familial B cell chronic lymphocytic leukemia, was found to be attributable to a germline SNP [c.1–6531 A>G] located upstream of the *DAPK1* promoter. Presence of this SNP resulted in higher binding affinity for the HOXB7 protein [19]. Similarly, hypermethylation of the *MLH1* and *MLH2* genes, also referred to as “epimutations” in inherited cases, have been associated with germline variants in some cases [20], [21]. In contrast, in Beckwith-Wiedemann syndrome, an *IGF2* gene polymorphism has been associated with loss of imprinting of the maternal allele-specific methylation of the *KCNQ1* gene [22]. The association observed in the current study between particular inherited *VHL* haplotypes and promoter hypermethylation in renal tumors may be an example of facilitated epigenetic variation, or an increased inherited propensity towards epigenetic variation with respect to a particular genotype [23]. Because we did not analyze *VHL* promoter hypermethylation in germline DNA (or in normal somatic tissues), it is unknown whether this particular finding is an example of an epimutation, however additional studies are warranted. This observation could be important for future translational research, as identification of individuals with high risk haplotypes could benefit from increased surveillance. Also of note, haplotype analyses spanning a 3-SNP sliding window across the *VHL* gene suggested that two regions may be driving the association observed, one centered within the *VHL* promoter and the other located 3′ of the *VHL* stop codon. The first region of interest, which spanned the promoter region of the *VHL* gene, raises the possibility that SNP variants in the promoter region could directly influence gene expression [24]. The second region associated with promoter hypermethylation in tumor DNA was centered 3′ to the *VHL* stop codon. It is possible that this region could be in linkage disequilibrium with a cis-acting structural or copy number alteration that might permit a mechanistic explanation to the associations observed [25].

*VHL* inactivation in tumor tissue was not associated with any of the clinical parameters examined that are normally considered to reflect disease progression such as stage, grade, and the presence of metastases or positive lymph nodes. The lack of an association between *VHL* alteration prevalence and indicators of tumor progression was similar to some previous reports [26]–[31]. However, this finding was in contrast to our previous report of 205 sporadic cases [6], and a recent study (analyzing 177 patient tumors) that reported an association between *VHL* promoter hypermethylation and tumor grade [13]. The mutational spectrum observed in our study was similar to others reporting that the majority of genetic alterations in RCC were located in exon 1 (codons 54–114) (an excellent summary of recent studies is provided in ref 13). The prevalence of promoter hypermethylation observed among cases in our study was within the range observed in other studies (∼5–15%), and was lower than that observed in a recent large study (a prevalence of 31% compared to ∼9% in our study) [13]. Unlike Young et al., [13] we did not observe a difference in the prevalence of alterations between tumors from male and female cases. These dissimilarities could be partially explained by differences in the laboratory methods used to detect promoter hypermethylation and/or the case selection criteria used in each study. Our case series came from a hospital-based case-control study that attempted to include all cases diagnosed at hospitals serving a specific geographic region per study center, and therefore may be more representative of the general study population of sporadic cases than those acquired through studies that uniquely include cases from tertiary care centers.

We observed a higher prevalence (∼8%) of *VHL* wild type cases among current smokers compared to never smokers in this study. The higher prevalence of *VHL* wild type cases among smokers had been previously observed in our study of 205 cases but was not statistically significant (p = 0.07). A Dutch study also observed more *VHL* wild type tumors among smokers [16]. A Swedish study did not observe more *VHL* wild type tumors among smokers overall, however, they observed that smoking modified the associations observed between *VHL* alteration prevalence and citrus fruit and vegetable intake [9]. Unlike the Swedish study, we observed an association between *VHL* promoter hypermethylation and low fruit intake frequency among smokers, rather than genetic alteration prevalence. One reason for these differences may be the use of formalin fixed tissues in both studies, compared to the use of frozen tissue in the current study.

The 8% difference in the prevalence of *VHL* wild type tumors among current smokers compared to never smokers could be clinically meaningful as this subgroup is considered biologically distinct from those with an inactivated VHL protein. Molecular studies have shown that *VHL* wild type and *VHL* inactivated tumors demonstrate differential signaling pathways [32]–[35] and methylation profiles [36]. Clinical studies have reported mixed results with respect to disease progression and survival. *VHL* wild type tumors have shown reduced treatment response rates [37] and lower median progression free survival [35]. In contrast, other studies have reported that cases without *VHL* alterations had better cancer-free and overall survival [34], [35], [37]–[41], with the exception of Stage IV disease, as found in one study [39]. However, two studies reported no differences [13], [27]. Clearly, elucidation of the association between heterogeneous tumor subtypes with respect to progression and survival warrants further large studies of well-characterized cases to enable pooled analyses across studies. Follow-up of cases in the current study is ongoing.

Occupational exposure to TCE was associated with an increased risk of RCC in this case-control study [42], however we were unable to replicate previous findings from a German study of exposed workers in which a higher *VHL* mutation prevalence and a hotspot located at nt454 (codon 81) was reported [11]. In the current study, only one unexposed case had a *VHL* mutation located at this location and the *VHL* mutation prevalence was similar in TCE exposed and unexposed cases. Although the German study result may have been a false-positive finding due to small sample size, it was unlikely that mutations were caused by artifacts introduced by formalin fixation, as each tumor DNA sample was analyzed in duplicate [11]. Another possibility was that the German workers may have been exposed to higher TCE levels than those in the current study. A second study of RCC tumor heterogeneity recently conducted among TCE-exposed workers in France, also did not replicate the high mutation prevalence and hotspot observed in tumors from the German exposed workers [43]. However, the French study reported an unusually low *VHL* alteration prevalence overall, which may have been due to the sensitivity of the laboratory methods applied and the use of DNA extracted from formalin fixed rather than frozen tumor material, as frozen tissue generally results in higher yields of good quality DNA. Both differences could cause misclassification of cases by their alteration status which would have biased results toward the null.

Strengths of the current study include a large sample size, a high participation rate, histological confirmation of all cases and also the tumor tissue used for DNA extraction. The study also included a high number of subjects from most case centers. We applied accurate and sensitive, mutation detection methods in a large study of well-characterized RCC and ccRCC cases to provide a clear picture of *VHL* inactivation through sequence alteration and promoter hypermethylation in a study conducted in a region with the highest incidence of RCC world-wide [44]. CGH analysis of chromosome 3p and specifically the clone harboring the *VHL* locus enabled us to evaluate the *VHL* region for biallelic loss. The multivariate analysis provided additional evidence to support that the only patient characteristic or risk factor associated with 3p or *VHL* locus loss was inactivation of the *VHL* gene through either genetic or epigenetic mechanisms, and the association was highly statistically significant. To our knowledge, this study is one of the largest conducted to date. However, additional large studies, data pooling, and meta-analyses will be required to clarify many of the associations observed across study populations. Lastly, we did not observe an increased risk associated with smoking in this case-control study as would have been expected; however by conducting case-only analyses, biases caused by control selection were eliminated.

Some weaknesses of this study included misclassification of fruit, vegetable, and alcohol intake frequency due to retrospective recall using a limited 23-item food frequency questionnaire. There may also have been misclassification of BMI and self-reported hypertension among cases, as this information was collected post- rather than prior to diagnosis. These qualities are strengths of the ongoing Dutch cohort study. Lastly, the prevalence of *VHL* gene epigenetic inactivation in tumor tissue was only about 9%. Therefore, this analysis relied upon few cases that were both heterozygous/homozygous for the *VHL* germline variants which also had promoter hypermethylation in tumor tissue. This resulted in unstable point estimates, and is reflected by some of the wide confidence intervals observed.

In summary, this comprehensive analysis of 470 well-characterized ccRCC patient tumor DNA samples observed *VHL* inactivation through genetic or epigenetic mechanisms in 86.6% of cases. The remaining 13.4% of cases in which we did not observe evidence of *VHL* inactivation (*VHL* wild type cases) may represent a biologically distinct subgroup, one that was observed more frequently in tumor DNA from smokers than never-smokers. Moreover, common germline *VHL* SNPs and haplotypes were associated with promoter hypermethylation in RCC tumor tissue and may demonstrate an example of facilitated epigenetic variation with respect to inherited high risk genotypes [23]. These findings show for the first time in a well-defined ccRCC case series that somatic *VHL* gene alterations in tumors were uniquely associated with exposures (i.e. tobacco smoking, diet) and inherited *VHL* polymorphisms in germline DNA, rather than factors associated with disease progression. Additional work is required to elucidate the consequences in these *VHL* molecularly defined subtypes in terms of etiology, biological mechanisms, treatment, and survival.

## Methods

### Ethics Statement

The study protocol was approved by relevant ethics committees and institutional review boards of all participating centers, the International Agency for Research on Cancer (IARC), and the U.S. National Cancer Institute (NCI) at the U.S. National Institutes of Health. All study subjects and their physicians provided written informed consent.

### Study Population

Cases were from a hospital-based case-control study of sporadic RCC that was conducted between 1999 and 2003 in seven centers in four countries of Central and Eastern Europe (Moscow, Russia; Bucharest, Romania; Lodz, Poland; and Prague, Olomouc, Ceske-Budejovice, and Brno, Czech Republic) as previously described [45], [46]. All newly diagnosed and histologically confirmed cases of sporadic kidney cancer (ICD-O2 code C.64) were identified at participating hospitals in each study region between 1999 and 2003. Histological slides of renal tumor tissue from all cases were reviewed by an international renal cancer pathology expert at the U.S. National Cancer Institute for standardized confirmation and disease classification (MM). Only confirmed cases of RCC and ccRCC were included in the analyses. There were 1097 cases included in the final case-control study: 524 of the 1097 cases (48%) originally diagnosed with RCC from hospital reports that provided frozen tumor biopsies for genetic analysis. Of the 524 cases, 507 (97%) cases were confirmed with RCC by review of their biopsy material provided, and 470 of the 507 (93%) were confirmed with the ccRCC subtype.

Questionnaires were administered in person by trained interviewers at each center. Subjects were asked about their lifestyle habits, in particular tobacco consumption, anthropometric measures one year before diagnosis, personal and familial medical history, and dietary habits. Lifetime occupational information for jobs of at least 12 months duration was also collected during interviews through the use of general occupational and job-specific questionnaires. Job-specific questionnaires covered (1) possible organic and chlorinated solvent exposures, (2) hours per week of solvent exposure, (3) source of solvent exposure, and (4) a description of solvent use as previously described [42]. All coding in the re-assessment was performed while blinded with respect to the previous assessment (with respect to chlorinated solvents and TCE exposures) as well as disease status.

Frequencies of fruit, vegetable, and alcohol intake were examined as they have been inversely associated with the prevalence of *VHL* alterations and particular alteration subtypes [9]. The dietary questionnaire was comprised of 23 food items which the study investigators selected by consensus during the planning stage of the study, which had been validated as previously described [47]. The questionnaire was repeated for two different time periods: 1) the year prior to interview, and 2) prior to political and market changes in 1989 (1991 in Russia). A lifetime weighted average intake for the two time periods was calculated as previously described [47].

### Tumor DNA Samples

Frozen tumor biopsies were collected from a subset of cases enrolled in the case-control study in which detailed information on tumor pathology, patient characteristics, and occupational risk factors for RCC, had been collected (Table S1). Tumor DNA extraction was performed following an additional pathology review of each tissue sample (FW) followed by macrodissection to remove non-tumor tissue, as previously described [6]. Sample areas estimated to contain at least 70% tumor cells were removed for DNA extraction. For each sample, 5 mm^3^ of tissue was sectioned and digested with 0.4 µg Proteinase K per µl of digestion buffer (500 mM KCl, 100 mM Tris-HCl, 15 mM MgCl_2_, 0.5% Tween 20) at 50°C overnight. A standard protocol^1^ was used to extract DNA from the digested samples.

### Analysis of *VHL* Sequence Alterations in Tumor DNA

PCR of *VHL* coding sequences, endonuclease scanning, and sequencing were performed as previously described [6]. PCR primers for this study for exons 1–3 were the following: *VHL* Exon1- Forward:5′-CTACGGAGGTCGACTCGGGAG, *VHL* Exon 1- Reverse:5′-GGGCTTCAGACCGTGCTATCG (amplicon length:495 bp); Exon 2- Forward:5′-CCGTGCCCAGCCACCGGTGTG, Exon 2- Reverse:5′-GGATAACGTGCCTGACATCAG (amplicon length:288 bp), Exon 3- Forward:5′-CGTTCCTTGTACTGAGACCCTAG, Exon 3- Reverse:5′-GAACCAGTCCTGTATCTAGATCAAG (amplicon length:317 bp).

### PCR

Amplification was carried out in 50 µL reactions using 10 to 15 ng tumor DNA and HotMaster Taq DNA Polymerase (Eppendorf). Thermal cycling was accomplished using a MJ Research (Bio-Rad) DNA Engine and a touchdown PCR program with an annealing temperature of 58C. PCR products were heteroduplexed using standard conditions. PCR products were analyzed on 2% agarose gels and electrophoresis.

### Endonuclease Scanning

Heteroduplexed PCR samples were analyzed using SURVEYOR Nuclease (Transgenomic, Inc.) and standard non-denaturing HPLC conditions appropriate for DNA fragment sizing as previously described [6]. A 100-bp DNA ladder (New England Biolabs) was run as a size marker. Positive and negative controls were included with each plate of PCR products to monitor endonuclease cleavage efficiency.

### Sequencing

Excess PCR primers were removed using the AMPure PCR Purification system (Agencourt Bioscience Corp.). Reaction chemistry using BigDye version 3.1 (Applied Biosystems) and cycle sequencing on an MJ Research thermal cycler were adapted from the manufacturer's recommendations. Sequencing products were purified using CleanSEQ reagents (Agencourt Bioscience). Sequence chromatograms were analyzed using Sequencher (GeneCodes, Ann Arbor, MI).

### *VHL* Promoter Methylation

Standard methods were used for bisulfite modification of 100 to 500 ng of tumor DNA (Zymo Research Laboratories). Primers were designed to amplify both methylated and unmethylated alleles across 11 CpG dinucleotides of the *VHL* promoter. Primers for the methylation analysis were the following: wild-type (WT) amplicon: *VHL* WT-Forward-CTACGGAGGTCGACTCGGGAG, WT-Reverse-GCGATTGCAGAAGATGACCTG (amplicon length:335 bp); *VHL* nested primers: WT Forward-CGGGTGGTCTGGATCG, WT-Reverse AGTTCACCGAGCGCAGCA (nested amplicon length:226 bp). Post-bisulfite modification primers that annealed to both methylated (M) and unmethylated (U) alleles were as follows: *VHL* M/U Forward-5′-TTAYGGAGGTYGATTYGGGAG, and *VHL* M/U Reverse- ACRATTACAAAAAATAACCTA, (amplicon length:335 bp), nested primers *VHL* M/U Forward-YGGGTGGTTTGGATYG, VHL M/U Reverse AATTCACCRAACRCAACA, nested (amplicon length: 226 bp). PCR was performed using an MJ Research PTC200 thermal cycler and a touchdown PCR program with an annealing temperature of 50°C. Nested PCR included 1 µL of a 1∶10 dilution of first-round product using cycling conditions as described above. PCR products were visualized in 2.0% agarose and bi-directionally sequenced. Cytosine positions in CpGs were inspected for thymine or cytosine signals in chromatograms, and scoring was conducted as follows: T only, not methylated; both cytosine and thymine, partially methylated; C only, fully methylated. Tumor samples with at least four of 11 methylated CpGs (>36%) were considered as hypermethylated. All analyses were run in duplicate, blinded to *VHL* mutation status, and with positive (CpGenome Universal Methylated DNA, Chemicon/Millipore) and negative (K562 Human Genomic DNA, Promega) controls.

### Array CGH

Each DNA sample from the same case-series was analyzed using Scanning and OncoBAC arrays. Scanning arrays were comprised of 2464 BACs selected at approximately megabase intervals along the genome as described previously [48], [49]. OncoBAC arrays were comprised of 960 Pa, PAC or BAC clones. About three-quarters of the clones on the OncoBAC arrays contained genes and STSs implicated in cancer development or progression. All cones were printed in quadruplicate. DNA samples for array CGH were labeled as described previously [48], [49]. Briefly, 500 ng each of cancer and normal female genomic DNA sample was labeled by random priming with CY3- and CY5-dUTP, respectively; denatured; and hybridized with unlabeled Cot-1 DNA to CGH arrays. After hybridization, the slides were washed and imaged using a 16-bit CCD camera through CY3, CY5, and DAPI filters [50]. Array CGH data image analyses were performed as described previously [51], [52].

### Tag SNP Analysis of Inherited *VHL* Polymorphisms

Genomic DNA was extracted from blood samples as described previously [42]. Single nucleotide polymorphisms (SNPs) were selected from the HapMap Project, using expected minor allele frequencies reported among Caucasians, to capture 80–90% of common genetic variation across the locus encompassing the *VHL* gene, covering an average of 15 kb upstream and downstream of the gene. This method relies upon linkage disequilibrium (LD) of highly correlated SNPs in order to identify gene regions that are associated with disease susceptibility, or in this case, specific heterogeneous subgroups of cases defined by their *VHL* alteration status [14]. SNPs selected (from 5′ to 3′ included the following: rs6442154 (*c3orf10*, Ex3 −90 T>C), rs6796538 (*VHL*, −5011 A>G), rs265318 (*VHL*, −2872 A>C), rs779805 (*VHL*, Ex1 +19 A>G), rs779812 (*VHL*, IVS1 −1184 G>A), rs1678607 (*VHL*, IVS2 +108 G>T), rs1642742 (*VHL*, Ex3 +473 A>G), rs1642739 (*VHL* 3835 bp 3′STP C>A), rs457414 (*IRAK2*, −3754 A>C), rs11465853 (*IRAK2*, IVS1 +249 G>C). Assays were validated at the NCI Core Genotyping Facility (CGF) in 280 control samples from the human diversity panel that included 76 African/African Americans, 66 Caucasians, 49 Native Americans/Hispanics, and 89 Pacific Rim Asians. Subsequently, minor allele frequencies (MAFs) were determined from the CGF panel among Caucasians. All selected SNPs had a minor allele frequency of at least 10%. Methods for assays can be found at: http://snp500cancer.nci.nih.gov. DNA samples were blinded and randomized on PCR plates to avoid any potential bias and duplicate genotyping was performed for a randomly selected 5% of the total series for quality control. Concordance rates were >99% for all SNPs. All SNPs analyzed were within the expected distributions of Hardy-Weinberg equilibrium (p<0.05).

### Statistical Analyses

Tumor and patient characteristics including clinical stage, Fuhrman nuclear grade (I–IV), node stage (N0, N1, N2–3), body mass index (BMI) (<25, 25–35, >35), and smoking status were considered as categorical variables. Smoking status (never, former, or current smoker) was defined as status 2 years prior to the interview. Specifically, participants who were smoking in the 24 months prior interview were classified as current smokers. Other variables such as metastasis (M0, M1) self-reported hypertension (no/yes), family history of cancer or kidney cancer (no/yes), sex, and age at diagnosis (<50, ≥50 years) were analyzed as dichotomous variables. Additional exposures and risk factors were selected based upon previous published reports of renal tumor heterogeneity including our own [6]. These additional factors included fruit, vegetable, and alcohol intake frequency, and *VHL* tagging SNPs [9]–[13]. Because occupational trichloroethylene (TCE) exposure was positively associated with RCC risk in this population [42] and because TCE exposure had previously been associated with an increased prevalence of *VHL* mutation among occupationally exposed workers [11], [12], we also evaluated associations between occupational exposure to organic and chlorinated solvents and trichloroethylene with *VHL* alteration prevalence. For nutritional variables, intake frequencies of related foods were summed to form food group intake categories based on tertile cutoff points that were defined by consumption frequency among controls. Categories of consumption for food-specific items were grouped as low (never to <once per month), medium (≥once per month but <once per week), and high (≥once per week to daily). Categories of alcohol consumption as grams per week of ethanol among weekly drinkers were none, low (<36.5), medium (36.5–137.5), and high ≥137.5) as described previously [47].

The prevalences of *VHL* genetic (sequence alterations) and epigenetic alterations (promoter hypermethylation) observed in tumor DNA samples were considered as dichotomous variables per case (no/yes). *VHL* gene nonsynonymous sequence alterations were considered as inactivating alterations if they were located within exons 1–3 and would lead to an altered amino acid sequence or a truncated VHL protein. Splice site mutations and promoter hypermethylation were also considered as inactivating alterations. DNA sequence alterations that were synonymous or SNPs were not considered as inactivating alterations. Similarly, sequence alterations that were located 5′ of codon 54, which were very rare, were not considered as inactivating alterations unless they were nonsynonymous and also would cause an alteration in the coding sequence of both pVHL19 and pVHL30 translation products. These included alterations such as insertions, deletions, and nonsense mutations. As in our previous study, the P25L variant was only considered an inactivating alteration if the case also possessed a second *VHL* alteration that would be considered inactivating using the above criteria. The prevalence of cases with a particular type of alteration was calculated by dividing the number of cases with that type of alteration by the total number of cases analyzed in the group. Chi-square tests were applied to contingency table (2×2) analyses to test for differences between the proportion of cases with or without a particular alteration subtype within each group. Trend tests were used to analyze associations between categorical variables and cases with particular types of alterations. Ordered logistic regression was used for multivariate analyses to evaluate associations between categorical variables and case subgroups, initially adjusting for variables that were associated with the same alteration subtype in our univariate analyses (p<0.20; Table S2). With the exception of sex, age, and country, other variables remained in multivariate models if their inclusion modified risk estimates by at least 10%.

For genotyping analyses, each SNP was assessed in three categories, (G_0_ = homozygote for the major allele, G_1_ = heterozygote, G_2_ = homozygote for the minor allele) using the most common allele as a referent. LD between SNPs was measured using Lewontin's D' statistic. Correlation (r^2^) between tagged regions was evaluated in Haploview. To evaluate associations between SNPs and *VHL* promoter hypermethylation prevalence, logistic regression models were used to calculate odds ratios (OR) and 95% confidence intervals (CI), adjusting for sex, age, country, and fruit intake. Risks were estimated using both additive and dominant models. Risk per allele and trends were calculated using unconditional logistic regression. Analyses were conducted using STATA 10.0 (Stata Corporation, College Station, TX) and all statistical tests were two-sided.

Haplotype analysis was used to explore the combined contribution of consecutive *VHL* tagging SNPs. A sliding window analysis was first conducted in MatLab to identify regions of interest (The MathWorks, Inc., Natick, MA). For the 10 SNPs examined, sliding windows of 2–9 SNPs were evaluated, accounting for multiple testing and correlations between SNPs. The significance of haplotype-based associations was assessed using the score test [53]. Haplotypes of interest were analyzed using an R package Haplostats (Version 1.3.1) in (version 2.4.1) adjusting for sex, age, and country. The most common haplotype was used as the reference group and rare haplotypes with frequencies of <5% were combined into one group. Generalized linear models accounting for phase ambiguity were used to estimate haplotype-specific ORs per copy [54], [55].

For array CGH analyses of these same cases, those with loss of chromosome arm 3p or clone CTB110j24 to those without, the presence of a loss was considered as a dependent variable in stepwise logistic regression models to first evaluate associations with clinical risk factors including: T stage (T1, T2, T3–4), Fuhrman nuclear grade (1–2, 3–4), node positivity (N0, N1, N2–3), and presence of metastases (M0, M1, Mx). In addition, co-variates and etiologic risk factors (described above) that were previously associated with RCC risk were initially analyzed using univariate analyses, including the presence of a *VHL* inactivating alteration in tumor tissue (no/yes-any). The criterion for initial inclusion of a variable into multivariate models was a p-value<0.20. Frequency matching variables inherent to the study design (i.e. country, sex and age) were included into all of the models regardless of associations with outcome. Variables selected were then fitted in logistic models adjusted for sex, country, and age, to obtain odds ratios (OR) and 95% confidence intervals (95% CI) as estimates of risk for both types of alterations. All analyses were conducted using SAS 9.1.3 software (SAS Institute Inc.) and STATA 10.0 and statistical tests were two-sided.

## Supporting Information

Table S1 Distribution of patient and ccRCC tumor characteristics among cases included and not included in VHL alteration study.
(XLS)

Table S2 Univariate analysis of von Hippel-Lindau inactivating alterations associated with patient descriptive and clinical characteristics in clear cell RCC cases.
(XLS)
